# ActivityDiff: a diffusion model with positive and negative activity guidance for *de novo* drug design

**DOI:** 10.1093/bioinformatics/btag564

**Published:** 2026-08-06

**Authors:** Huimin Zhu, Renyi Zhou, Jing Tang, Min Li

**Affiliations:** School of Computer Science and Engineering, Central South University, Changsha 410083, China; School of Computer Science and Engineering, Central South University, Changsha 410083, China; Research Program in Systems Oncology, University of Helsinki, Helsinki 00290, Finland; School of Computer Science and Engineering, Central South University, Changsha 410083, China

## Abstract

**Motivation:**

*De novo* drug design requires not only promoting desired target activity, but also avoiding undesired target interactions that compromise selectivity and safety. However, existing generative models largely focus on optimizing positive activity, while overlooking negative activity information that could help suppress off-target effects during molecular design.

**Results:**

We present ActivityDiff, a classifier-guided diffusion framework for activity-controlled molecular generation. Unlike conventional approaches that rely primarily on positive activity optimization, ActivityDiff explicitly incorporates both positive and negative guidance through separately trained drug–target classifiers. This design enables the model not only to promote desired target activities, but also to suppress harmful off-target interactions during generation. Experiments show that ActivityDiff effectively supports various drug design tasks, including single- and dual-target generation, fragment-constrained dual-target design, selective generation for improved target specificity, and reduction of off-target effects. These results demonstrate that classifier-guided diffusion with explicit negative guidance provides an effective strategy for jointly optimizing efficacy and safety in molecular design.

**Availability and implementation:**

The source code can be obtained from https://github.com/e-yi/ActivityDiff.

## 1 Introduction

A drug’s biological behavior critically influences its efficacy and safety. Rational drug design therefore involves balancing multiple objectives, including maximizing on-target efficacy, minimizing toxic off-target effects, and, when needed, optimizing activity across multiple therapeutic targets (Csermely *et al.* 2005, Li *et al.* 2021, Ravikumar and Aittokallio 2018). Such multi-target modulation is particularly important in complex diseases, including cancer and neurodegenerative disorders, where improved therapeutic outcomes often depend on coordinated regulation of multiple disease-relevant targets (Geerts *et al.* 2020). Accordingly, effective molecular design strategies should support flexible optimization of multi-target activity while limiting off-target risk.

Deep learning models show significant potential in drug–target interaction prediction (Lu *et al.* 2025) and drug design (Imrie *et al.* 2020, Li *et al.* 2021, Peng *et al.* 2022, Uludogan *et al.* 2022, Xia *et al.* 2026), leading to improved efficiency and reduced development costs. Some methods are primarily used to generate molecules that meet specific chemical properties (Mahmood *et al.* 2021, Wu *et al.* 2024). Other generative methods have demonstrated great potential in navigating the complex drug-like chemical space, thereby improving the efficiency of discovering molecules with therapeutic activity (Kotsias *et al.* 2020, Zhang *et al.* 2023). However, generative methods typically focus on single-target activity generation. For example, Pocket2mol (Peng *et al.* 2022) utilizes the pocket structure as a condition to generate molecules that bind the pocket. KGDiff (Qian *et al.* 2023) uses a network that predicts Vina score to guide the generation process and generates molecules with improved docking score. DeepDTAGen (Shah *et al.* 2025) is a multi-task deep learning model designed to simultaneously predict drug–target binding affinities and generate new drug candidates. These methods are typically confined to single-objective drug design and cannot generate compounds under multi-target activity constraints.

A few approaches have been proposed to address multi-target drug design with activity constraints. A straightforward strategy involves connecting fragments known to be active against different targets, such as DeLinker (Imrie *et al.* 2020), SyntaLinker (Yang *et al.* 2020), DEVELOP (Imrie *et al.* 2021), and DiffLinker (Igashov *et al.* 2024). However, fragment linking approaches often suffer from the drawback of generating molecules with excessively large molecular weights. Alternatively, other *de novo* methods have been developed using different design strategies. AIxFuse (Chen *et al.* 2024) learns pharmacophore fusion patterns to satisfy dual-target structural constraints in molecular docking simulations. POLYGON (Munson *et al.* 2024) is a multi-pharmacology method based on generative reinforcement learning that predicts compounds capable of inhibiting multiple proteins while maintaining high drug similarity and synthetic feasibility by embedding chemical space. DRAGONFLY (Atz *et al.* 2024) integrates protein–protein interactions, drug–target interactions, and drug–drug relationships to construct an interaction network, and employs a graph neural network to generate molecules conditioned on this network.

In addition to optimizing activity against intended targets, it is also crucial to suppress unintended interactions with other targets, thereby improving binding selectivity and reducing the risk of off-target effects and toxicity. Analyses of clinical failures from 2010 to 2017 reveal that while 40%–50% of candidates fail due to insufficient efficacy, 30% are abandoned for safety issues, among which unanticipated off-target interactions represent an important contributing factor (Sun *et al.* 2022). For example, torcetrapib, a CETP inhibitor, was terminated in late-stage clinical trials due to off-target activation of the mineralocorticoid receptor, which led to increased cardiovascular events (Niesor *et al.* 2015, Vergeer *et al.* 2008). These studies highlight the urgent need for methods that not only improve target affinity but also explicitly suppress harmful off-target effects (Zhao *et al.* 2025).

In this work, we propose ActivityDiff, a diffusion-based molecular generative framework aimed at enabling selective, off-target minimized, and multi-target molecular generation. It uses pre-trained drug–target interaction classifiers to guide each step of the reverse diffusion process, providing fine-grained control over molecular generation toward predefined pharmacological objectives. Through positive prompting, the model is encouraged to generate molecules with high affinity for desired targets. Negative prompting is introduced to suppress unintended affinities toward off-target proteins, thereby reducing the risk of binding-related toxicity. Moreover, ActivityDiff supports multi-target generation by allowing simultaneous application of multiple prompts, enabling both multi-target generation and selective generation between closely related targets. We evaluate the model’s controllability and generalizability through diverse generation experiments, including single-target design, dual-target *de novo* generation, fragment-constrained dual-target generation, selective generation to improve target specificity, and generation minimizing off-target activity. In all cases, the model consistently produces molecules that align with the desired activity profiles specified by the classifiers, demonstrating strong adaptability to different design objectives.

## 2 Materials and methods

### 2.1 Dataset

The diffusion model is trained on GEOM (Axelrod and Gomez-Bombarelli 2022). In the experiments, we consider atom types (H, B, C, N, O, F, Al, Si, P, S, Cl, As, Br, I, Hg, Bi, Se, Fe, Pt, V, Rh, Co, Ru, Mg), six formal charge states {−2, −1, 0, +1, +2, and +3}. We use kekulized molecules, so there are four edge types (none, single bond, double bond, and triple bond) to be considered.

The classifiers are trained on drug–target interaction data from BindingDB (Huang *et al.* 2021). For each target, compounds are split into training, validation, and test sets at an 8:1:1 ratio. Activity labels are assigned as follows: compounds with activity <1000 nM are labeled as active, those with activity ≥10 000 nM as inactive, and compounds with intermediate activity values (1000–10 000 nM) are assigned continuous labels in [0,1] using log-scale interpolation for smoother classifier supervision. To improve generalization, negative sampling is applied by randomly selecting inactive compounds with structural similarity <0.6 to active compounds. The final active-to-inactive ratio in the training set is 1:10.

### 2.2 ActivityDiff architecture

ActivityDiff is a controllable molecular generation framework built on discrete diffusion and classifier guidance. Its key advantage is that generation and guidance are partially decoupled, so new design objectives can be introduced without retraining the generative model. Unlike conventional classifier-guidance methods that mainly promote desired properties, ActivityDiff introduces bidirectional and compositional guidance, allowing the model to both enhance desired properties and suppress undesired ones while supporting multi-objective molecular design.

**Figure 1:**
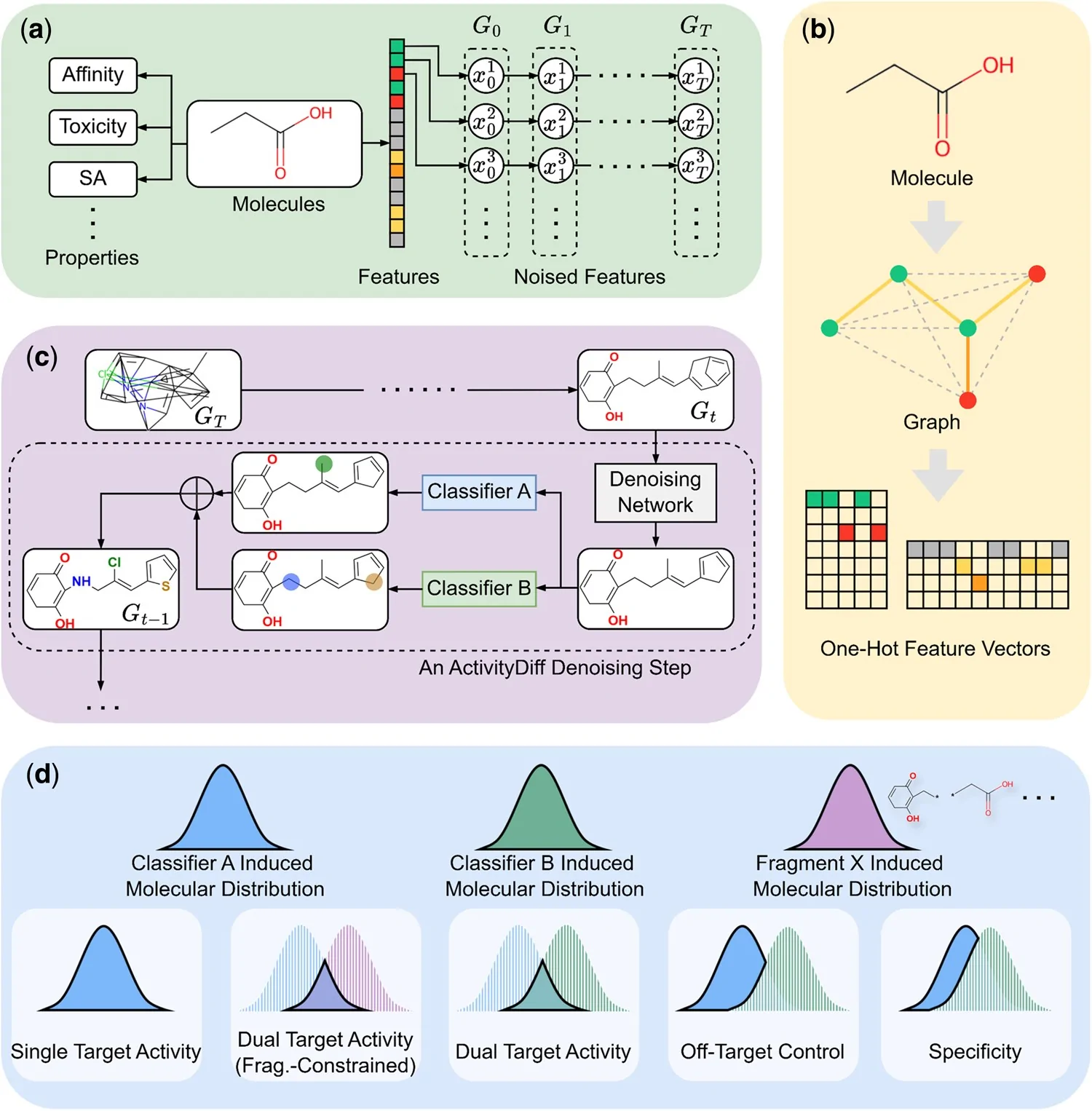
An illustration of ActivityDiff. (a) Dependency graphical model of ActivityDiff. (b) Graph representation of a molecule as a complete graph with node and edge feature vectors. (c) Denoising process of ActivityDiff. (d) Representative downstream applications of ActivityDiff.

As shown in Fig. 1, ActivityDiff represents a molecule as a graph composed of atoms and bonds, which are encoded as discrete node and edge features (Fig. 1a and b). During the forward process, these features are progressively corrupted with noise, and the model learns to recover valid molecular structures through iterative denoising. During generation, separately trained property classifiers evaluate noisy intermediate molecules and provide guidance signals at each denoising step, steering the generation toward desired properties or away from undesired ones (Fig. 1c). By changing the guidance setup, ActivityDiff can be applied to diverse drug design tasks, including single-target generation, fragment-constrained design, multi-target optimization, selectivity optimization among homologous proteins, and off-target avoidance (Fig. 1d). Details of the diffusion model and classifier architectures are described below.

#### 2.2.1 Discrete denoising diffusion

We represent a molecule with N atoms as an undirected complete graph G={V, E}, where $V=\{v_{i},\;i=1,2,\ldots ,N\}$ is the set of vertices with each vertex characterized by its atom symbol and formal charge, and $E=\{e_{j},\;j=1,2,\ldots N,\;\frac{N\times (N-1)}{2}\}$ is the set of edges with each edge characterized by its bond type (including non-bond) and the nodes it connects.

To enhance controllability during molecular generation, we adopt a discrete denoising diffusion model, which consists of a forward process that adds noise and a reverse denoising process. Following D3PM’s (Austin *et al.* 2021) proposed framework, for a discrete random variable with K categories of the graph, x∈{1,2,…,K}, the forward process q progressively corrupts it and yields a sequence of corrupted variables ${\left\{x_{t}\right\}}_{t=0}^{T}$, where $x_{0}$ represents original discrete features and $x_{T}$ aligns with the maximally corrupted state distribution π. Let $x_{t}$ be represented by a one-hot column vector, and the forward process at each step t can be described by a categorical distribution $q\left(x_{t}|x_{t-1}\right)=\mathrm{Cat}(x_{t};\;P=Q_{t}x_{t-1})$, where $Q_{t}\in R^{K\times K}$ is the transition matrix, Cat(.) represents the categorical distribution.

We apply such a forward process independently to each categorical feature in the graph G during the corruption process of a molecule, such that $q\left(x_{t}|G_{t-1}\right)=q\left(x_{t}|x_{t-1}\right)$, where $G_{t}$ represented the noisy graph at time step t. In the reverse process p, the aim is to reconstruct the original $G_{0}$ from its corrupted state $G_{T}$ by progressively denoising it, which is formulized as follows (Ho *et al.* 2020, Hoogeboom *et al.* 2021):


(1) $$p_{\theta }(x_{t-1}|G_{t})=\sum_{x_{0}}q(x_{t-1}|x_{0},x_{t})p_{\theta }(x_{0}|G_{t})$$

where $q(x_{t-1}|x_{0},x_{t})$ is the posterior distribution induced by the forward process, and $p_{\theta }(x_{0}|G_{t})$ is the estimated marginal distribution of $x_{0}$ given $G_{t}$. In practice, the denoising network is trained to directly predict $x_{0}$ from the noisy graph $G_{t}$ using the cross-entropy objective:


(2) $$\mathrm{loss}\_\mathrm{diff}=E_{t{,G}_{0},G_{t}}[-\log p_{\theta }(x_{0}|G_{t})]$$

#### 2.2.2 Classifier blocks

We choose the MPNN from dgllife (Li *et al.* 2021) as the structure of the classifiers. It contains a graph neural network encoder consisting of several layers of MPNN, a Set2Set pooling layer and a MLP to produce the final prediction. This model leverages atomic features and formal charges as node attributes, and bond types as edge features. Molecular graphs are encoded via graph convolutional layers that aggregate information from neighboring nodes through a message-passing mechanism, enabling the model to learn expressive node-level representations. These representations are subsequently aggregated and passed through linear layers for final classification. Each classifier is trained as a binary target-activity classifier to distinguish active and inactive molecules for a given target.

During classifier training, noise is added to input molecules at randomly sampled diffusion steps to simulate intermediate diffusion states. The trained classifier is used to score noisy molecules during generation, with losses at different noise levels weighted by the signal-to-noise ratio (SNR) to preserve informative supervision under high noise conditions. Specifically, a weighted binary cross-entropy loss with SNR-based weighting is used:


(3) $$\mathrm{loss}\_\mathrm{cls}=-\frac{1}{N}\sum_{i=1}^{N}[y_{i}*\log(\sigma (z_{i}))+(1-y_{i})*\log(1-\sigma (z_{i}))]*{\mathrm{snr}\_\mathrm{weight}}_{i}$$

where $z_{i}$ is the model logit, $y_{i}$ is the ground-truth label, ${\mathrm{snr}\_\mathrm{weight}}_{i}$ denotes the SNR-based weight for the sample *i*, and *N* is the number of samples.

#### 2.2.3 Classifier-guidance with discrete denoising diffusion

Independently trained classifiers are leveraged to steer the generation process toward molecules with desired characteristics. The key advantage of this approach is its flexibility: because the classifiers are trained separately from the generator, ActivityDiff can be adapted to specific scenarios without retraining the entire generative model, significantly reducing adaptation costs.

During the reverse process, the objective is modified to incorporate the condition y, where y represents the desired molecular feature. The overall conditional objective becomes $p(G_{t-1}|G_{t},y)$ and for each variable x, the objective be rewritten using Bayes’ rule and the dependence relations as:


(4) $$p(x_{t-1}|x_{t},y)\propto p_{\theta }(x_{t-1}|x_{t})p_{\phi }(y|G_{t-1})$$

where $p_{\theta }(x_{t-1}|x_{t})$ is the same with unconditional generation, and $p_{\phi }(y|G_{t-1})$ is a separately trained classifier that predicts the likelihood of the condition y being satisfied given the noisy molecular graph $G_{t-1}$ at time step $t-1.\;p_{\phi }(y|G_{t-1})$ is then approximated by performing a first-order Taylor expansion around a reference graph $G_{t-1}^{*}$:


(5) $$p_{\phi }(y|G_{t-1})\approx p_{\phi }(y|G_{t-1}^{*})+(G_{t-1}-G_{t-1}^{*})\cdot \nabla_{G_{t-1}}p_{\phi }(y|G_{t-1}){|}_{G_{t-1}=G_{t-1}^{*}}]$$

Positive guidance increases the predicted activity for desired targets, whereas negative guidance decreases the predicted activity for undesired or off-target proteins. Since $G_{t-1}\;$contains both atom and bond features, this signal guides both nodes and edges at each reverse denoising step. This approximation allows the reverse process to favor molecular graphs that better satisfy the target condition.

### 2.3 Implementation details

The model is trained using the Adam optimizer with a learning rate of 0.0003 and no weight decay for up to 1000 epochs. The denoising network contains 6.00M parameters and each classifier 0.68M parameters. Early stopping with a patience of 30 is applied based on validation AUC. Diffusion model training was performed on a single Tesla V100S-PCIE-32GB GPU, while all other experiments were conducted on a single NVIDIA Tesla V100 GPU with 32 GB memory. Training required approximately 66 h, and generating 10 000 molecules took approximately 5.5 h of wall-clock time (≈2 s per molecule). Detailed computational costs for classifiers across different targets are provided in Table 1, available as supplementary data at *Bioinformatics* online. An archived version of the code associated with this manuscript is available at Zenodo: https://doi.org/10.5281/zenodo.20412080.

**Table 1:** Molecular generation performance of ActivityDiff and other models.

Methods	Validity	Uniqu-eness	Novelty	VUN score
ORGAN	0.379	0.841	0.687	0.219
VAE	0.870	0.999	0.974	0.847
SMILES LSTM	0.959	1.000	0.912	0.875
Syntalinker	1.000	0.880	0.903	0.795
PGMG	0.948	0.999	1.000	0.948
LS-MolGen	0.632	1.000	0.761	0.481
Pocket2Mol	0.999	0.349	0.997	0.348
DEVELOP	0.856	0.334	0.997	0.286
REINVENT2.0	0.963	0.999	0.999	0.961
**ActivityDiff (unconditional)**	**0.978**	**0.999**	**0.999**	**0.975**
**ActivityDiff (conditional)**	**0.796**	**0.999**	**0.999**	**0.795**

The bold values in Table 1 indicate the results obtained using the method proposed in this study. Specifically, ActivityDiff (unconditional) refers to generation without classifier guidance, whereas ActivityDiff (conditional) refers to generation with classifier guidance.

## 3 Results

### 3.1 Performance of ActivityDiff in generation experiments

We benchmark the performance of ActivityDiff in molecular generation settings against established baselines, including SMILES-based methods such as VAE (Gómez-Bombarelli *et al.* 2018), ORGAN (Guimaraes *et al.* 2017, 2017), SMILES LSTM (Segler *et al.* 2018), Syntalinker (Yang *et al.* 2020), PGMG (Zhu *et al.* 2023), LS-MolGen (Li *et al.* 2023), and REINVENT2.0 (Blaschke *et al.* 2020). Graph-based methods such as Pocket2Mol, DEVELOP (Imrie *et al.* 2021). The results of Pocket2Mol, DEVELOP, LS-MolGen, and REINVENT2.0 are from the MoGE benchmark (Zhang *et al.* 2025). The results of VAE, ORGAN, and SMILES LSTM are taken from GuacaMol (Brown *et al.* 2012).

As shown in Table 1, unconditional ActivityDiff achieves the highest VUN score (defined as the product of validity, uniqueness, and novelty), while achieving comparable levels of novelty, validity, and uniqueness to other top-performing models, including Syntalinker, REINVENT2.0, PGMG, and SMILES LSTM. See Supplementary Information, available as supplementary data at *Bioinformatics* online for analysis of invalid molecule types. The reduced validity under conditional generation is expected due to the additional constraints introduced by classifier-guided sampling, while uniqueness and novelty remain extremely high. These results indicate that ActivityDiff effectively balances novelty and distributional fidelity, demonstrating a strong capacity to generate diverse yet chemically plausible molecules.

### 3.2 Generating active-like molecules under classifier guidance

**Figure 2:**
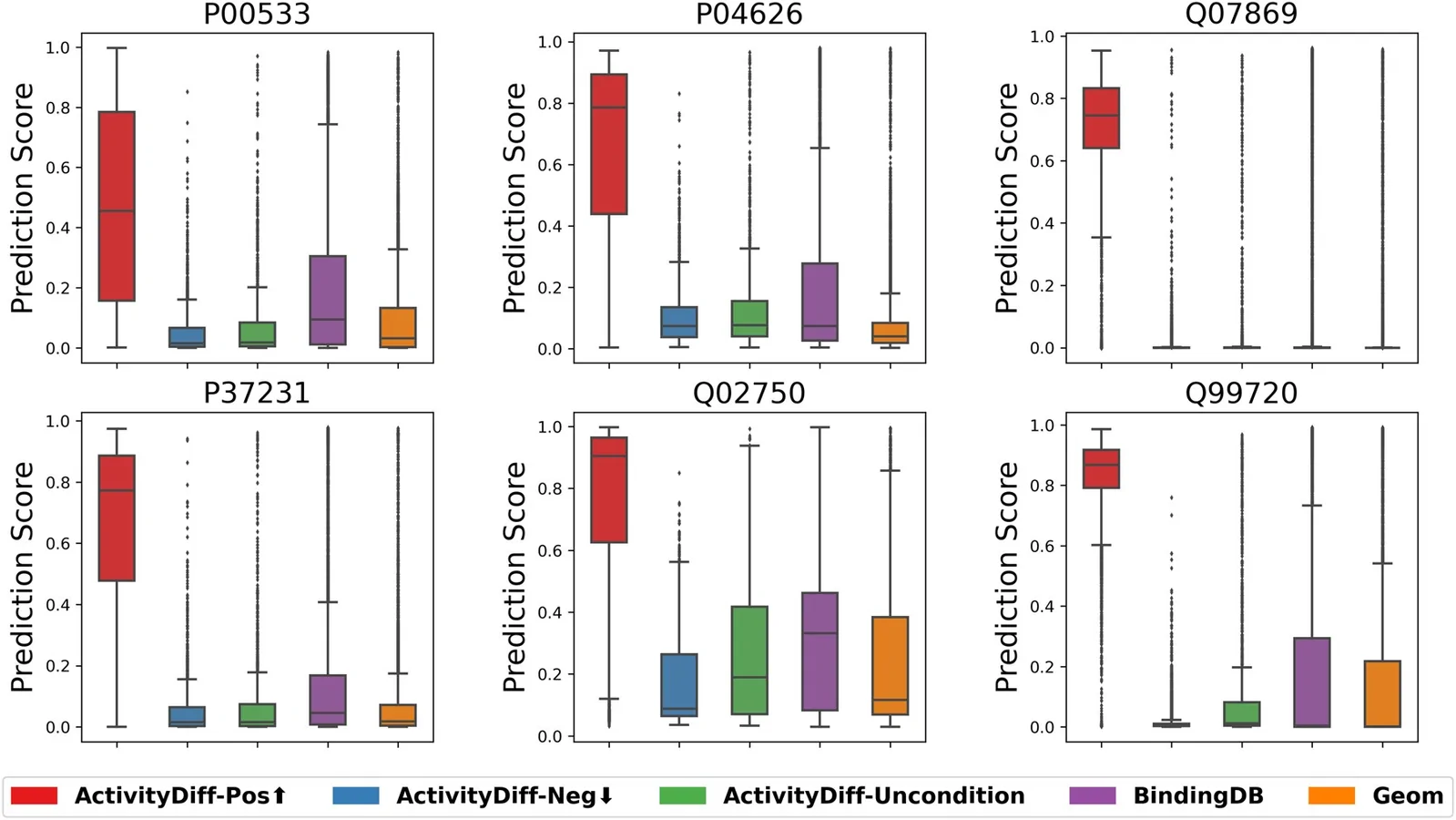
Boxplots showing the prediction scores based on the trained classifiers for molecules generated across different targets.

We evaluate the performance of these classifiers in guiding molecular generation, considering both positive and negative guidance scenarios. Positive guidance aims to generate molecules predicted to be active against the target, whereas negative guidance seeks to generate molecules predicted to be inactive. ActivityDiff is evaluated across six biological targets with detailed target information provided in Table 2, available as supplementary data at *Bioinformatics* online. For each target, 10 000 molecules are generated under each guidance mode, and three control groups are established: (i) 10 000 molecules randomly sampled from the generator’s training set (GEOM); (ii) 10 000 molecules randomly sampled from the classifier’s training set (BindingDB); and (iii) 2000 molecules generated without guidance using ActivityDiff’s base diffusion model, which is sufficient to approximate the distribution of the full generation set while reducing computational cost. Classifier performance across all targets is provided in Table 3, available as supplementary data at *Bioinformatics* online. All generated and control molecules are evaluated using the trained classification models to predict their activity probabilities, with results shown in Fig. 2.

**Table 2:** Docking performance of molecules generated by ActivityDiff and experimental molecules.

Target	Experiment	Top 1000
P00533 (1M17)	−8.336 ± 0.811	−9.638 ± 0.535
P04626 (3PP0)	−9.467 ± 1.864	−11.051 ± 0.439
Q07869 (1K7L)	−8.558 ± 0.799	−8.863 ± 0.495
P37231 (1I7I)	−8.994 ± 1.080	−9.241 ± 0.499
Q02750 (1S9J)	−8.434 ± 1.031	−9.155 ± 0.461
Q99720 (5HK1)	−9.426 ± 1.250	−10.908 ± 0.546

The results indicate that molecules generated under positive guidance exhibit a substantially higher proportion of predicted activity values exceeding 0.8 (Fig. 2) compared to the control groups. Specifically, the positive guidance group achieves 68.6% ± 11.9% (*n* = 6) of molecules in the high-activity region (*Y*** ≥ **0.5), whereas the GEOM dataset yields 4.9% ± 4.0% (Fig. 2), the BindingDB dataset 16.6% ± 6.0% (Fig. 2), and the unconditional generation group only 1.2% ± 1.0% (Fig. 2). These findings demonstrate that classifier-guided generation effectively biases molecular output toward the desired activity space. To further assess bidirectional controllability, we evaluated the effect of negative guidance. Molecules generated under negative guidance yielded the lowest predicted activity scores among all groups, with a mean score of 0.07 (SD 0.11), significantly lower (*P* < .001) than those from BindingDB (mean 0.22, SD 0.27), GEOM (mean 0.10, SD 0.18), unconditional generation (mean 0.11, SD 0.17), and positive guidance (mean 0.63, SD 0.31). Notably, 85.7% of molecules generated under negative guidance have classifier scores below 0.1, compared with only 6.5% in the positive-guidance group. Together, these results demonstrate that ActivityDiff enables robust bidirectional control over molecular generation.

We conduct docking experiments to offer an additional perspective on our results and confirm the reliability of the selected candidates. For each target, we generate 10 000 molecules using ActivityDiff with single positive guidance, and we use AutoDock Vina (Trott and Olson 2010) to predict the docking scores of those molecules. We show the top 1000 molecules generated by ActivityDiff. As shown in Table 2, the results indicate that a large proportion of the molecules generated for different targets are identified as being within the range of active molecules in terms of docking scores.

**Figure 3:**
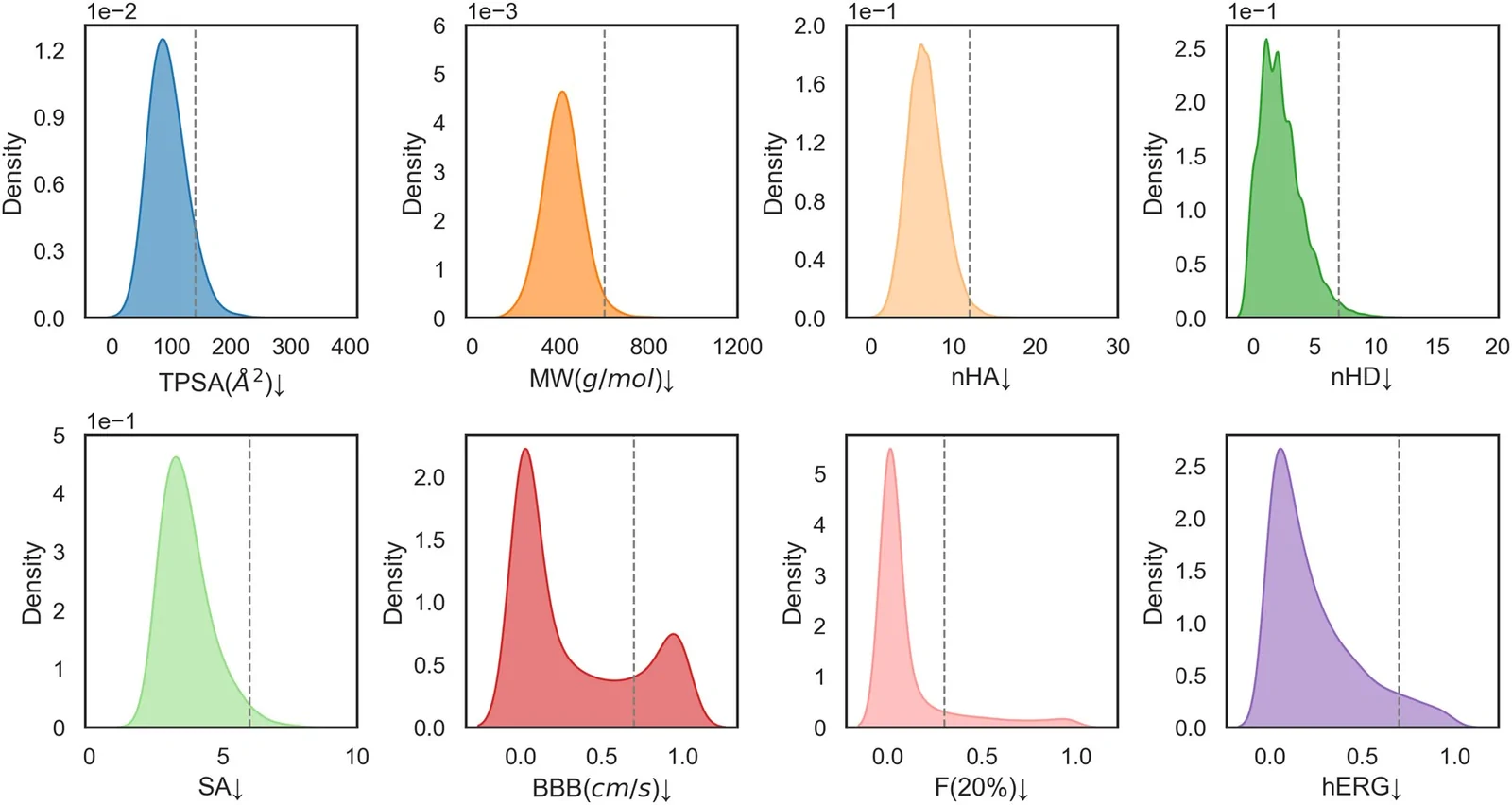
Distributions of the ADMET properties of the top 1000 molecules generated by ActivityDiff. Dashed lines indicate the recommended thresholds for each property; downward arrows denote properties for which lower values are preferred. TPSA (0–140 Å^2^), MW (100–600 Da), nHA (0–12), nHD (0–7), and SA (<6) describe molecular physicochemical and synthetic properties; BBB (0–0.7) indicates blood–brain barrier penetration probability, and *F* (20%) (<0.3) indicates a low probability of oral bioavailability <20%. hERG denotes predicted probabilities related to cardiotoxicity.

To assess the drug-likeness of molecules generated by ActivityDiff, we evaluated their pharmacokinetic profiles—including absorption, distribution, metabolism, and excretion—as well as predicted toxicity for the top 1000 generated compounds. As illustrated in Fig. 3, the majority of these molecules fall within acceptable ranges for key ADMET properties, meeting commonly used criteria for drug candidates as defined by ADMETlab 2.0 (Xiong *et al.* 2021).

### 3.3 Case study

#### 3.3.1 Generating dual-target compounds

**Figure 4:**
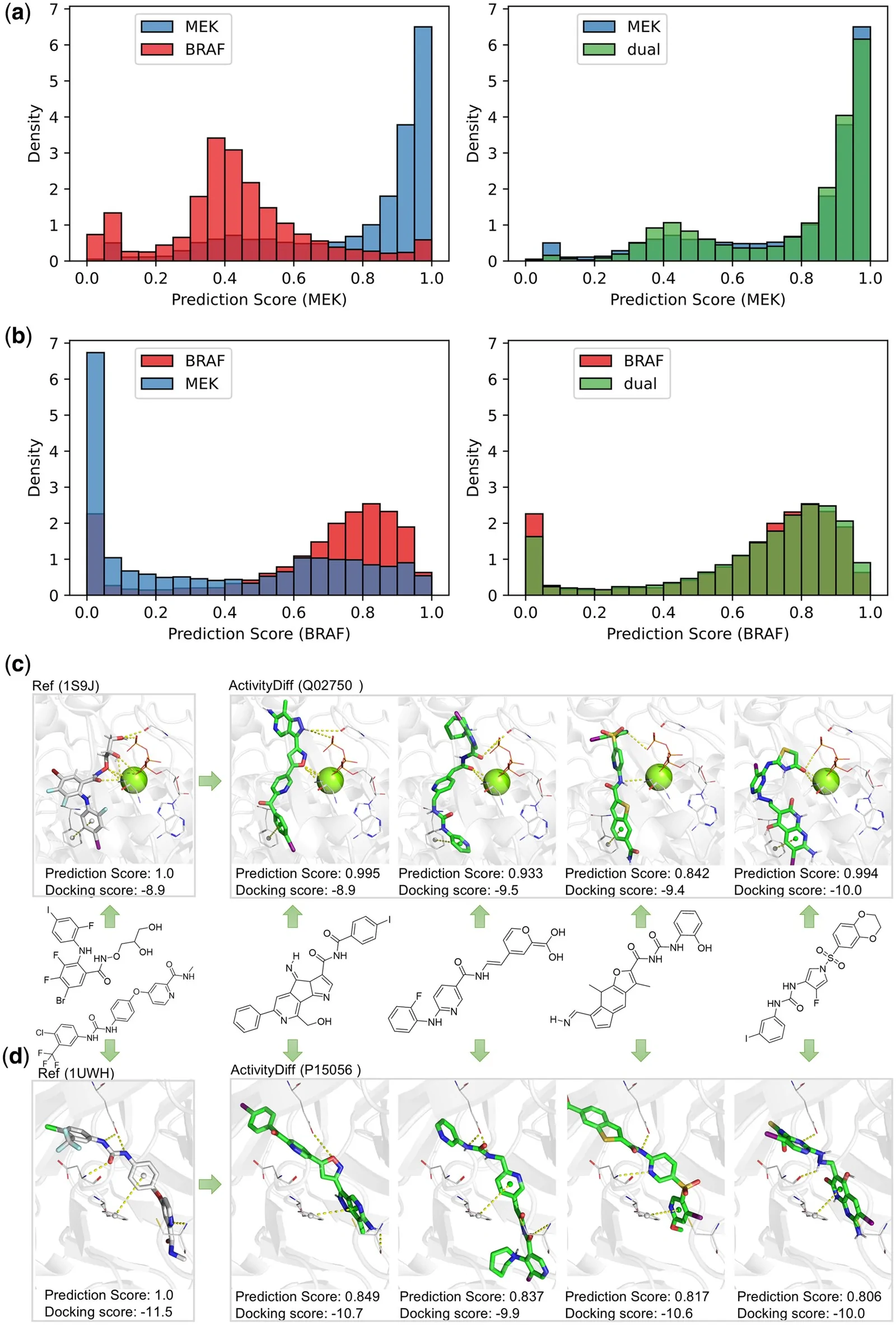
(a) MEK prediction scores for molecules generated under different guidance strategies: single target guidance (left); combined BRAF–MEK versus MEK guidance (right). (b) BRAF prediction scores for molecules generated under different guidance strategies: single target guidance (left); combined BRAF–MEK versus MEK guidance (right). (c) MEK binding analysis of molecules generated using dual guidance: the prediction scores (MEK classifier), docking scores and binding conformations of reference (left, PDB: 1S9J) and generated molecules (right). (d) BRAF binding analysis of molecules generated using dual guidance: the prediction scores (BRAF classifier), docking scores and binding conformations of reference (left, PDB: 1UWH) and generated molecules (right). Dashed lines indicate ligand–protein interactions.

Melanoma is often associated with mutations in the NRAS and BRAF genes within the MEK signaling pathway. Research has shown that the combination of BRAF/MEK inhibitors show significant synergy for BRAF-mutant cancers (Greco *et al.* 2019). In this case, we train two classifiers to identify active and inactive molecules targeting MEK and BRAF. The classifiers are then used in the ActivityDiff generation process to guide molecular generation. Considering potential conflicts between the gradients of different targets during dual-guidance generation, we test two strategies: (i) directly summing the gradients from both classifiers, and (ii) projecting the gradient of the MEK classifier onto BRAF. The results shown in Fig. 4 correspond to the first approach.

Figure 4a shows the distribution of prediction scores by MEK classifier of molecules generated under single-target guidance and those generated using dual-classifier guidance. Specifically, molecules generated via BRAF-specific guidance (red) are evaluated using the MEK classifier, yielding predicted activity values predominantly within the 0.2–0.6 range (median = 0.417** ± **0.044), which is significantly lower than those in the MEK single-target guidance group (blue, median 0.905** ± **0.064; *P* < .01). Notably, under the dual-target guidance mode (green), the generated molecules exhibit a MEK activity score distribution (median = 0.903** ± **0.055) that is statistically indistinguishable from the MEK single-target guidance group, indicating that the dual-guidance system effectively preserves MEK-targeted activity.

The distribution of BRAF prediction scores for molecules generated under single-target guidance and dual guidance strategies, as evaluated by the BRAF activity prediction model, is shown in Fig. 4b. The predicted values under MEK guidance are predominantly distributed in the low-activity range (0–0.3, median = 0.30), showing a significant difference from those under BRAF single-target guidance, which primarily fall within the 0.6–0.9 range (median = 0.743; *P* < .01). Notably, when molecules are generated under BRAF/MEK joint guidance (Fig. 4b, green), the median BRAF activity score reaches 0.754, exhibiting a high degree of distribution overlap (>90%) with the BRAF single-target guidance group (median = 0.743), as quantified by the Bhattacharyya coefficient (Kang and Wildes 2015).

The binding sites of reference ligands from PDB and dual-target molecules generated using the joint BRAF–MEK classifier are shown in Fig. 4c and d for MEK (UniProt ID: Q02750, PDB: 1S9J) and BRAF (UniProt ID: P15056, PDB: 1UWH), respectively. The binding poses of generated molecules are obtained using molecular docking. Structural analysis reveals that classifier-guided molecules exhibit high compatibility with the MEK binding pocket and form well-aligned interactions within the BRAF binding site. Further examination indicates that both protein pockets engage in π–π stacking interactions and hydrogen bonding with the generated ligands, thereby enabling effective accommodation of both targets. These molecules achieve classifier scores exceeding 0.8 for both targets, and their docking scores are comparable to those of known protein–ligand complexes. In addition, we examined whether ActivityDiff could support dual-target design by combining fragment preservation for one target with classifier guidance toward another target, as described in Figs 1–3, available as supplementary data at *Bioinformatics* online. Collectively, these findings highlight the model’s capability to generate dual-target molecules.

#### 3.3.2 Generating specific inhibitors

HER2 (human epidermal growth factor receptor 2) and EGFR (epidermal growth factor receptor 1) are two highly homologous receptors with significant structural similarities. Although EGFR/HER2 combination inhibitors achieve favorable outcomes in the treatment of various cancers, they are often associated with adverse effects such as diarrhea and rashes, which can severely impact patients’ quality of life. In contrast, HER2-specific targeted drugs have shown distinct advantages in treating HER2-positive breast cancer (Swain *et al.* 2023). By selectively targeting HER2, these drugs can effectively inhibit tumor cell proliferation while avoiding EGFR-related side effects. In this study, the objective is to generate molecules with high activity toward HER2 (UniProt ID: P04626) while minimizing interactions with EGFR (UniProt ID: P00533). To this end, two classifiers are trained to recognize compounds with activity against EGFR and HER2, respectively. HER2 selectivity is promoted using positive guidance toward HER2 and negative guidance against EGFR. Considering that gradients from different classifiers may conflict, the EGFR classifier gradient is orthogonally projected onto the HER2 classifier gradient during generation, retaining only the HER2-aligned component to guide HER2-specific molecule generation.

**Figure 5:**
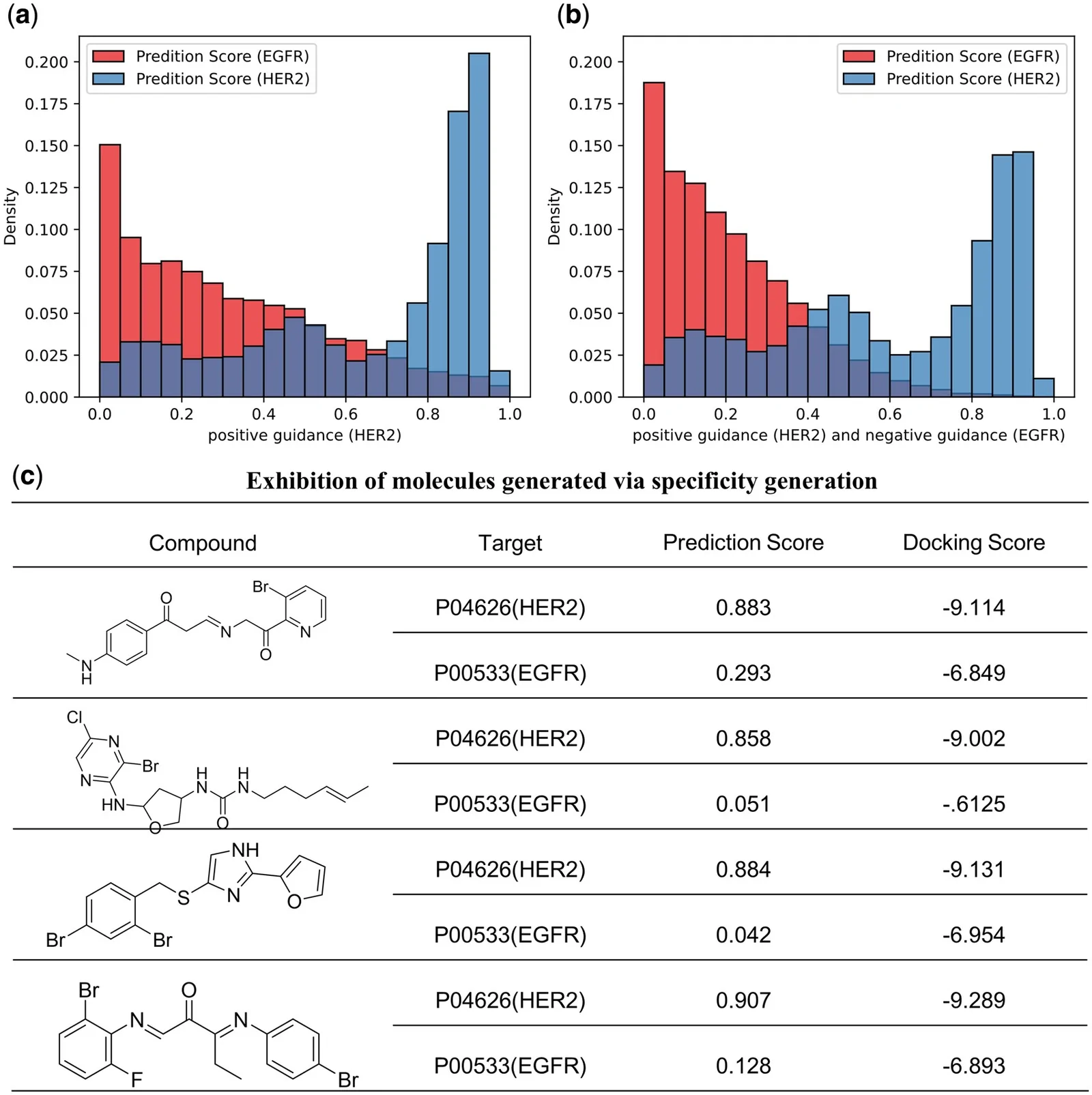
(a) The HER2 (UniProt ID: P04626) prediction scores and the EGFR (UniProt ID: P00533) prediction score of molecules generated by guiding with the HER2-positive guidance. (b) shows the HER2 prediction scores and the EGFR score of molecules generated using combined positive guidance from the HER2 classifier and negative guidance from the EGFR classifier. (c) Exhibition of molecules generated via specificity generation.

Figure 5a presents the prediction score distributions of molecules generated under HER2 classifier guidance, as evaluated by the HER2 classifier and the EGFR classifier. Figure 5b illustrates the distributions of molecules generated under combined HER2-positive and EGFR-negative guidance, also evaluated using the HER2 classifier and the EGFR classifier. Among molecules generated under HER2 classifier guidance, 22.7% exhibit EGFR prediction scores above 0.5 (Fig. 5a), suggesting potential dual affinity. However, this proportion decreases substantially to 6.4% (Fig. 5b) when using the specificity-guided approach, demonstrating the effectiveness of this strategy.

Notably, HER2 classifier scores remain high under specificity guidance. The proportion of molecules with HER2 scores above 0.5 is 69.3% with HER2 guidance and 62.2% with specificity guidance. Although a slight leftward shift is observed, likely due to the high homology between HER2 and EGFR, the generated molecules still show high HER2 and low EGFR scores, as well as favorable docking results (Fig. 5c). These findings indicate that ActivityDiff improves target specificity while preserving HER2 activity.

#### 3.3.3 Reducing broad-spectrum off-target effects

Studies have shown that off-target interactions are a major contributor to adverse drug effects (Sun *et al.* 2022). In preclinical drug development, off-target panels are widely used to assess candidate molecules (Bowes *et al.* 2012, Liu *et al.* 2024). Such screening helps identify well-targeted compounds early, reducing safety risks and increasing the likelihood of clinical success. In this section, six targets are selected based on the sensitivity and hit-rate analysis of the BioPrint1 (Krejsa *et al.* 2003) dataset conducted by Peters *et al.* (2012), which identified targets that are highly predictive of compound promiscuous binding. For example, approximately 70% of compounds binding to the 5-HT2B receptor are classified as promiscuous across the panel, whereas only 0.64% of compounds with no significant affinity (pIC50 < 3.5) are identified as promiscuous (Hamon *et al.* 2009). Therefore, these six targets are considered to constitute a reduced yet representative off-target profile. A joint classifier is trained using experimental activity data from these six targets. A training sample is labeled as positive if it exhibited high binding activity toward any of the six targets, whereas samples with binding affinity below 10 000 nM (IC50) to all six targets are labeled as negative.

The generation experiment aims to produce target-selective molecules while avoiding binding to targets in the off-target panel. The generated molecules are then evaluated using an external off-target predictor (Liu *et al.* 2024). For comparison, experimentally validated active molecules are also assessed using the same predictor. A classification threshold of 0.5 is applied, such that molecules with prediction scores exceeding 0.5 are considered likely to interact with at least one target in the off-target panel.

**Figure 6:**
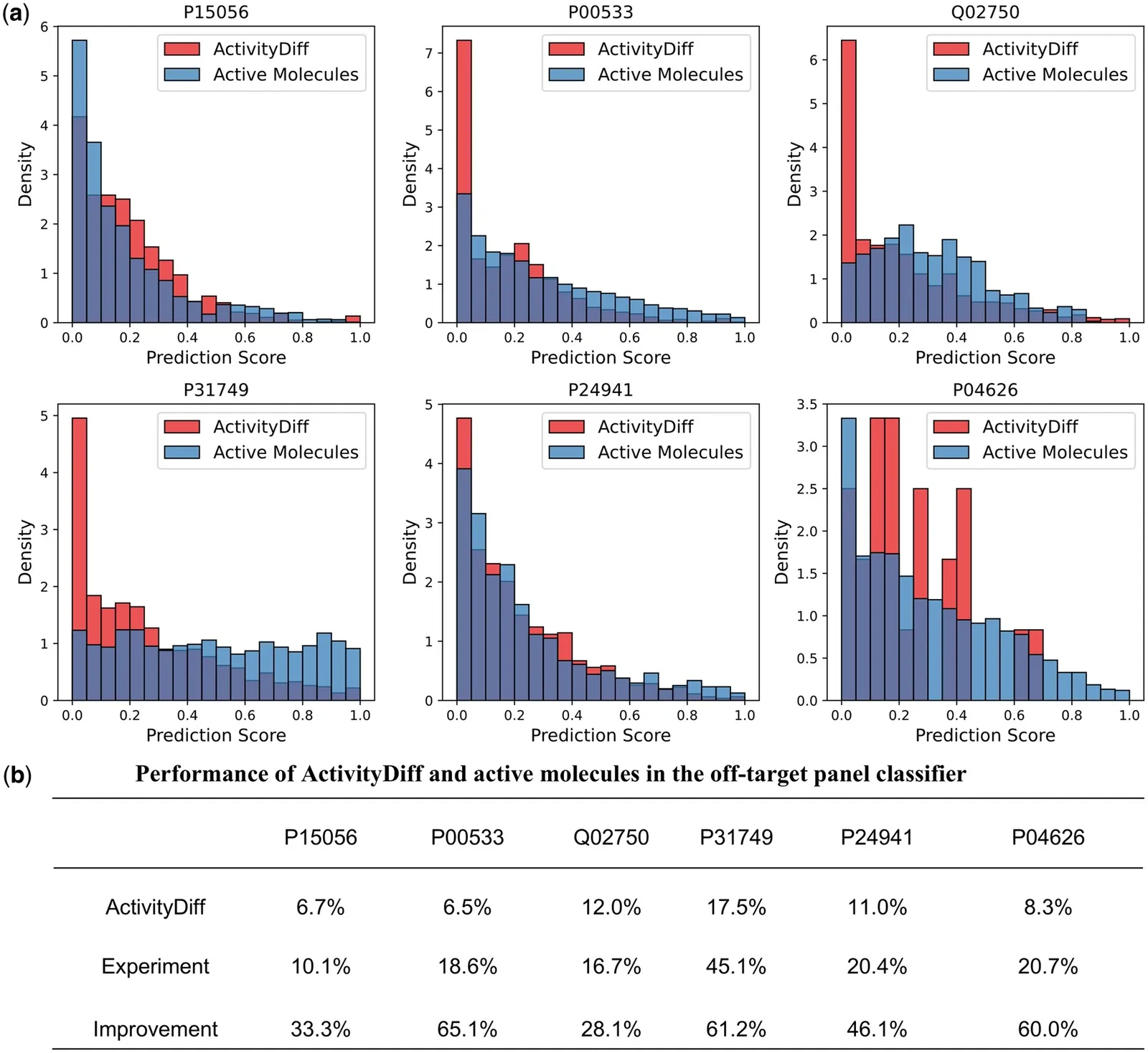
(a) Distribution of prediction scores in the off-target spectrum panel for ActivityDiff generated molecules and experimentally active molecules. (b) Performance of ActivityDiff and active molecules in the off-target panel classifier.

As shown in Fig. 6a, across all targets, molecules generated with the off-target panel classifier guidance exhibit a lower off-target ratio than the experimentally active molecules. Figure 6b shows the percentage of molecules predicted to carry off-target risk, based on the off-target prediction model, for both ActivityDiff-generated compounds and experimentally active molecules from BindingDB. “Improvement” reflects the reduction in off-target risk achieved by ActivityDiff relative to the experimental actives. As shown in the table, across the tested targets, compounds generated by ActivityDiff consistently show a lower proportion of predicted off-target liabilities than the experimental molecules.

## 4 Conclusion

In this study, we propose ActivityDiff, a framework built upon discrete diffusion models with classifier-based guidance, designed to suppress off-target risks, enhance selectivity, and support multi-target generation. The separately trained classifier reduces the cost of leveraging the method in a new scenario. The classifiers are leveraged to provide positive guidance, which improves desired properties, and negative guidance which reduces undesired off-target effects. The simplicity of positive and negative classifier guidance does not hinder the efficacy and flexibility of ActivityDiff.

The potential application of ActivityDiff spans diverse scenarios as demonstrated in our case studies. In single-target applications, it guides the generation of either active or inactive compounds, ensuring targeted optimization. When applied to multi-target drug design, ActivityDiff integrates multiple classifiers to facilitate the design of compounds with activity across different targets. Additionally, by incorporating negative guidance from undesired target classifiers, the approach enhances selectivity, minimizing off-target effects and toxicity. Furthermore, ActivityDiff’s use of fragments allows for the design of multi-target active molecules, offering a refined and effective strategy for complex molecular designs that balance both activity and selectivity.

Despite these strengths, several challenges remain. Balancing affinities across multiple targets, making better use of inactive compound data, and improving the prediction of metabolism and toxicity are all important for enhancing model reliability. Future work may therefore benefit from integrating drug design with systems-level biological networks that capture drug–protein, protein–protein, and drug–drug interactions.

## Supplementary Material

btag564_Supplementary_Data
