# Supplementary material for: ActivityDiff: a diffusion model with positive and negative activity guidance for de novo drug design
Source: Bioinformatics. 2026 Aug 6;42(8):btag564. doi: 10.1093/bioinformatics/btag564 (PMC13478866; doi:10.1093/bioinformatics/btag564)
Supplement: Supplementary file 1 [file btag564_supplementary_data.zip › ActivityDiff-Supplementary Information.docx]

**ActivityDiff: A diffusion model with Positive and Negative Activity Guidance for De Novo Drug Design**

Huimin Zhu^1,^^†^, Renyi Zhou^1,^^†^, Jing Tang^2^ and Min Li^1,*^

^1^School of Computer Science and Engineering, Central South University, Changsha 410083, China. ^2^ Research Program in Systems Oncology, University of Helsinki, Helsinki, 00290, Finland. ^†^These two authors contribute equally to the work.

*To whom correspondence should be addressed. Min Li: <limin@mail.csu.edu.cn>

**Computational Cost Analysis of Target Classifiers**

We evaluate the computational cost of training target-specific classifiers. The classifier training time scales approximately linearly with dataset size, with an average computational cost of approximately 1.2 seconds per molecule across different targets. Table S1 summarizes the dataset size and classifier training time for each target.

**Table S1. Training statistics of target-specific classifiers.**

| Target | Uniprot ID | Number of molecules | Classifier training time |
| --- | --- | --- | --- |
| EGFR | 1M17 | 40,030 | 14 h 05 min |
| ERBB2 | 3PP0 | 12,601 | 4 h 03 min |
| PPARA | 1K7L | 4,685 | 1 h 35 min |
| PPARG | 1I7I | 11,264 | 3 h 37 min |
| MAP2K1 | 1S9J | 5,032 | 1 h 42 min |
| SIGMAR1 | 5HK1 | 17,640 | 5 h 17 min |
| BRAF | 1UWH | 16,972 | 5 h 19 min |

**Targets Evaluated in ActivityDiff**

To evaluate the performance of ActivityDiff, we conduct comprehensive testing on a curated set of protein targets involved in diverse disease pathways, particularly those relevant to cancer, metabolic disorders, and neurological conditions. The selected proteins include members of the MAPK signaling cascade, nuclear hormone receptors, receptor tyrosine kinases, and serine/threonine kinases. Table S2 summarizes the detailed information of each target protein, including its UniProt ID, representative PDB structure, and biological relevance.

**Table S2. Summary of Proteins Evaluated in ActivityDiff**

| Target | Uniprot ID | PDB ID | Description |
| --- | --- | --- | --- |
| EGFR | P00533 | 1M17 | Receptor tyrosine kinase frequently altered in lung cancer and gliomas; |
| ERBB2 | P04626 | 3PP0 | HER2 proto-oncogene; overexpression drives breast and gastric cancers; |
| PPARA | Q07869 | 1K7L | Ligand-activated nuclear receptor; regulates lipid metabolism and links circadian rhythm to metabolism; |
| PPARG | P37231 | 1I7I | Nuclear receptor regulating lipid metabolism and adipogenesis; |
| MAP2K1 | Q02750 | 1S9J | MAPK cascade kinase; mutations associated with colorectal cancer; |
| SIGMAR1 | Q99720 | 5HK1 | Involved in lipid transport, cellular signaling, and neuroprotection. |

**ActivityDiff Classifier Performance Evaluation**

Here, we evaluate the performance of ActivityDiff to identify potentially active molecules with these targets. We first evaluate the performance of classifiers across different targets using three metrics: Area Under the receiver operating characteristic Curve (AUC), Average Precision (AP), and Best F1 Score. Table S3 shows the results**.**

The AUC values for all targets range from 0.936 to 0.968, indicating the classifiers performed exceptionally well in distinguishing between active and inactive molecules in the test set. Specifically, target Q99720 exhibits the highest AUC value of 0.968. In terms of AP, values range from 0.640 to 0.742, with Q02750, P00533 showing highest AP scores of 0.742, 0.735, indicating strong precision–recall performance in identifying active molecules for this target. The Best F1 Score revealed certain variability in performance. Overall, the classifier demonstrates strong predictive performance across multiple targets, with particularly outstanding results observed for P00533, and Q02750.

**Table S3. Evaluation of classifier's performance across different targets.**

| Target | AUC | AP | Best F1-score |
| --- | --- | --- | --- |
| P00533 | 0.949 | 0.735 | 0.700 |
| P04626 | 0.936 | 0.671 | 0.667 |
| Q07869 | 0.953 | 0.684 | 0.698 |
| P37231 | 0.956 | 0.640 | 0.660 |
| Q02750 | 0.955 | 0.742 | 0.678 |
| Q99720 | 0.968 | 0.735 | 0.694 |

**Analysis of Invalid Molecule Types**

To further investigate the sources of invalid molecules during molecular generation, we analyze the invalid outputs produced by both sequence-based and graph-based generative models, including the SMILES-based model PGMG and the graph-based models Pocket2Mol and ActivityDiff.

Interestingly, we observe that kekulization-related failures are substantially more common in the SMILES/sequence-based model, whereas graph-based models more frequently generate molecules with valence inconsistencies or chemically unreasonable bonding patterns.

This difference is likely related to the underlying generation mechanisms of the two model categories. In SMILES/sequence-based generation, molecules are generated autoregressively as token sequences, where aromaticity is implicitly encoded using aromatic atom symbols and ring closure syntax. As a result, syntactically valid SMILES strings may still contain inconsistent aromatic annotations or ring assignments, leading RDKit to fail during aromaticity perception or kekulization. In these cases, the generated structures are not necessarily completely random graphs, but rather chemically ambiguous aromatic systems that cannot be assigned a valid alternating single/double bond representation under RDKit sanitization rules.

In contrast, graph-based models directly generate atom types and bond connectivity on molecular graphs. Although this representation avoids some SMILES-specific aromatic syntax ambiguities, it introduces a different challenge: maintaining chemically valid local valence constraints during graph construction or denoising. Consequently, invalid molecules from graph-based methods are more frequently associated with explicit valence violations, such as atoms exceeding their permitted bonding capacities, rather than kekulization failures.

Therefore, the dominant sources of invalidity appear to differ between sequence-based and graph-based molecular generation frameworks. Our observations suggest that kekulization issues are not the primary invalidity source in graph-based generation models such as ActivityDiff and Pocket2Mol, where valence inconsistency remains the more common failure mode.

**Fragment-Constrained Dual-Target Generation**

Additionally, we evaluate ActivityDiff in a dual-target drug design setting, where molecules are generated toward one target while preserving an active fragment from another (Figure S1). This is achieved by constraining specific graph substructures during the diffusion process, ensuring that all generated molecules retain the designated fragment. Specifically, a core fragment is manually selected from an active molecule within the PDB complex of one target and used as a structural constraint, combined with classifier guidance toward the second target. As shown in Figure S1, the generated molecules successfully preserve the designated fragment while also achieving high prediction scores from the second target classifier.

Figure S1. Dual-target generation results by guiding molecule generation toward one target while preserving the active fragment of another. The left panel shows the binding sites of the two targets extracted from the PDB complex structures.

For each target, we selected one bioactive fragment from its ligand-bound complex structure and used it as a condition while guiding the generation process toward the other target, with the resulting molecules shown in the right panel. Additional molecules are provided in the Figure S2, Figure S3.

Figure S2. Molecules generated using Q02750-targeted classifier and P15056-targeted fragment.

Figure S3. Molecules generated using P15056-targeted classifier and Q02750-targeted fragment.
